# Pitfalls of Diffusion-Weighted Imaging: Clinical Utility of T2 Shine-through and T2 Black-out for Musculoskeletal Diseases

**DOI:** 10.3390/diagnostics13091647

**Published:** 2023-05-07

**Authors:** Yuri Kim, Seul Ki Lee, Jee-Young Kim, Jun-Ho Kim

**Affiliations:** 1 Department of Radiology, St. Vincent’s Hospital, College of Medicine, The Catholic University of Korea, Seoul 06591, Republic of Korea; yurikim0110@gmail.com (Y.K.); jeeykim@catholic.ac.kr (J.-Y.K.); 2 Department of Orthopaedic Surgery, Center for Joint Diseases, Kyung Hee University Hospital at Gangdong, Seoul 05278, Republic of Korea

**Keywords:** diffusion-weighted imaging, apparent diffusion coefficient, magnetic resonance imaging, pitfall, musculoskeletal diseases

## Abstract

Diffusion-weighted imaging (DWI) with an apparent diffusion coefficient (ADC) value is a relatively new magnetic resonance imaging (MRI) sequence that provides functional information on the lesion by measuring the microscopic movement of water molecules. While numerous studies have evaluated the promising role of DWI in musculoskeletal radiology, most have focused on tumorous diseases related to cellularity. This review article aims to summarize DWI-acquisition techniques, considering pitfalls such as T2 shine-through and T2 black-out, and their usefulness in interpreting musculoskeletal diseases with imaging. DWI is based on the Brownian motion of water molecules within the tissue, achieved by applying diffusion-sensitizing gradients. Regardless of the cellularity of the lesion, several pitfalls must be considered when interpreting DWI with ADC values in musculoskeletal radiology. This review discusses the application of DWI in musculoskeletal diseases, including tumor and tumor mimickers, as well as non-tumorous diseases, with a focus on lesions demonstrating T2 shine-through and T2 black-out effects. Understanding these pitfalls of DWI can provide clinically useful information, increase diagnostic accuracy, and improve patient management when added to conventional MRI in musculoskeletal diseases.

## 1. Introduction

Diffusion-weighted imaging (DWI) with apparent diffusion coefficient (ADC) values is a recent addition to the musculoskeletal magnetic resonance imaging (MRI) that has been adopted by many institutions. This is because some musculoskeletal diseases may have similar imaging findings of conventional MRI of T1- and T2-weighted images, which can limit the diagnostic specificity [1,2]. The primary role of DWI and ADC values in musculoskeletal radiology has been evaluated for tumorous diseases related to cellularity, such as the differentiation of benign and malignant tumors [3,4,5,6,7,8,9,10,11,12,13,14,15,16,17,18,19,20,21,22,23], determination of therapeutic response [24,25,26,27,28,29,30,31,32,33,34,35,36,37], and identification of recurrent disease [38,39]. Additionally, DWI has been used for non-tumorous diseases such as infection [40,41,42,43,44,45,46,47] and inflammation [48,49,50,51,52,53,54,55,56,57,58,59,60,61,62]. However, DWI has some pitfalls, including T2 shine-through and T2 black-out effects, due to the interaction between DWI and T2-weighted images [63]. Many review articles have included mentions of these pitfalls [64,65,66,67], but they have been noted as cautions rather than emphasizing their clinical usefulness. While these pitfalls should be interpreted with caution, they can be useful in diagnosing certain musculoskeletal diseases, as many lesions in the musculoskeletal system exhibit markedly hyperintense (i.e., ganglion cyst) or hypointense lesions (i.e., hematoma, fibrous tumor, fatty lesion, mineraliazation) on T2-weighted images. This review will focus on the DWI with ADC values in musculoskeletal radiology and their pitfalls including T2 shine-through [68,69,70,71] and T2 black-out effect [2,63,68,72], with clinical use in the diagnosis of musculoskeletal diseases.

## 2. Principles of DWI

DWI is a relatively new MRI sequence that provides qualitative and quantitative information on the cellularity of a lesion by measuring the Brownian motion of water molecules within the tissue [69,73,74]. The random motion of water molecules in tissue is the basis of the signal in DWI [75]. The greater the average free path length of a water molecule, the greater the signal loss achieved with diffusion-sensitizing gradients [1]. The first gradient of DWI causes the dephasing of the spins, while the second gradient rephrases the spins. If the molecules have moved before the second gradient is applied, the rephrasing is incomplete, and signal loss occurs, which can be visualized and measured as an ADC value [76]. Signal losses on DWI are proportional to both (i) the free motion of water molecules in tissues and (ii) the diffusion-sensitizing gradients used [1].

Point: DWI provides qualitative and quantitative information on the cellularity of a lesion by measuring the Brownian motion of water molecules within the tissue, and the greater the average free path length of a water molecule, the greater the signal loss achieved with diffusion-sensitizing gradients.

### 2.1. Water Movement in Tissues

Water movement in tissues is not completely free or random, but can be influenced by interactions with tissue microstructures, suggesting that DWI reflects differences in the Brownian motion of water molecules caused by changes in tissue microscopic structure [63]. DWI captures the extracellular, intracellular, and transcellular motion of water molecules as well as microcirculation (perfusion) [75]. Among these components, extracellular and perfusion contribute the most to the signal loss of DWI [75]. However, obstacles such as macromolecules, tight junctions, membranes, and membranous structures impede diffusion, resulting in a reduction in the motion of intracellular water molecules in biological tissue compared to the extracellular space [77]. Different patterns of water molecule diffusion in various biologic tissues help determine the contrast obtained with DWI, indicating not only cellularity but also fibrosis or hemoglobin degradation products [70]. In vascular structures and tissues that have lost cellular integrity (e.g., necrotic tissue), water molecules diffuse greater and show less signal on DWI. Tissues with highly cellular microenvironments have restricted water diffusion and exhibit high signals on DWI [63].

Point: DWI reflects differences in the Brownian motion of water molecules caused by changes in tissue microscopic structure, capturing the extracellular, intracellular, and transcellular motion of water molecules, while obstacles such as macromolecules, tight junctions, membranes, and membranous structures impede diffusion, resulting in a reduction in the motion of intracellular water molecules in biological tissue compared to the extracellular space.

### 2.2. Diffusion Sensitizing Gradients

**Figure 1:**
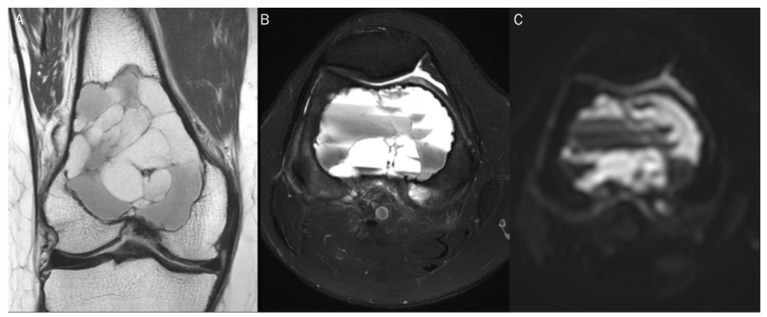
A 60-year-old female with giant-cell tumor with secondary aneurysmal bone cyst change in the distal femur. (**A**) Coronal T2-weighted image and (**B**) axial T2-weighted fat-suppressed image show secondary aneurysmal bone cyst change with fluid–fluid level. (**C**) The DWI with *b*-value of 0 s/mm^2^ appears similar to the heavily T2-weighted fat-suppressed image (**B**).

**Figure 2:**
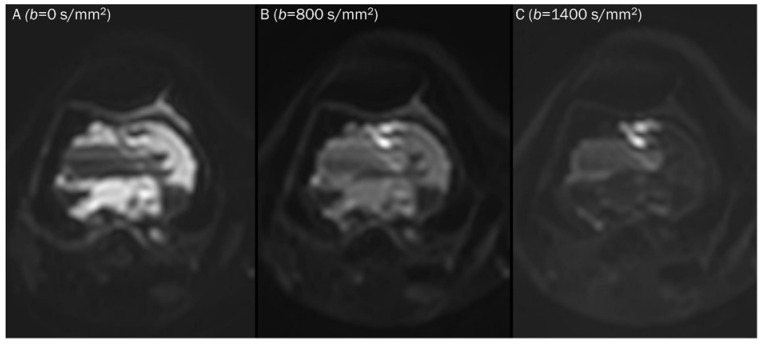
Progressive increase in *b*-values (same patient in Figure 1). As *b*-values increase on DWI (**A**–**C**), the perfusion effect is gradually suppressed and only hypercellular tissues remain bright at high *b*-value images with a darkening background.

DWI can measure water diffusivity by applying diffusion-sensitizing gradients to T2-weighed spin-echo sequences using echoplanar readouts of the data [2]. In the absence of a diffusion sensitizing gradient (*b*-value of 0 s/mm^2^), free water appears bright with its own T2-weighting (Figure 1). As the *b*-values increases (Figure 2), signal intensity decreases steadily in tissues, first in free water (e.g., urine in the bladder), then in glandular tissue (e.g., prostate, salivary gland, pancreas), and highly organized cellular structures such as the liver. Because highly packed tissue such as tumors restrict water movement, highly cellular tissues appear continuously bright against a darkening background at high *b*-values of 500–1000 s/mm^2^.

**Figure 3:**
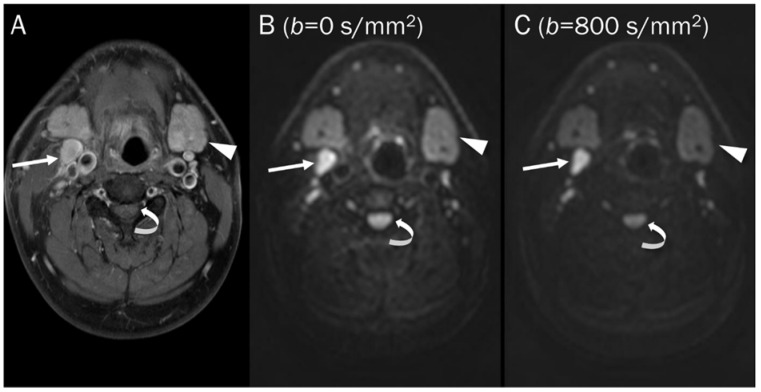
DWI of a pathologic lymph node, a normal submandibular gland, and a normal spinal cord. (**A**) Axial T1-weighted enhanced image of the neck shows a pathologic lymph node (arrow), a normal submandibular gland (arrowhead), and a normal spinal cord (curved arrow). These hypercellular tissues remain bright on DWI with increasing *b*-value images (**B**,**C**).

For similar reasons, several normal but highly cellularized tissues such as the brain, spinal cord, spleen, and normal lymphatic tissues, are also displayed as bright signals in high *b*-values (500–1000 s/mm^2^) images (Figure 3) [2].

Point: DWI measures water diffusivity by applying diffusion-sensitizing gradients to T2-weighed spin-echo sequences, with signal intensity decreasing steadily in tissues as *b*-values increase, except for highly cellular tissues such as tumors which appear continuously bright against a darkening background at high *b*-values.

## 3. Values in Obtaining DWI

The strength and duration of the diffusion-sensitizing gradients used in DWI are indicated by the “*b*-value”, and typically, radiologists select two or more *b*-values (expressed as s/mm^2^) to evaluate tissue water diffusivity [2]. The ADC value is calculated as the slope of the exponential decrease in signal intensity between DWIs acquired with different *b*-values [77].

### 3.1. b-Value Selection

**Figure 4:**
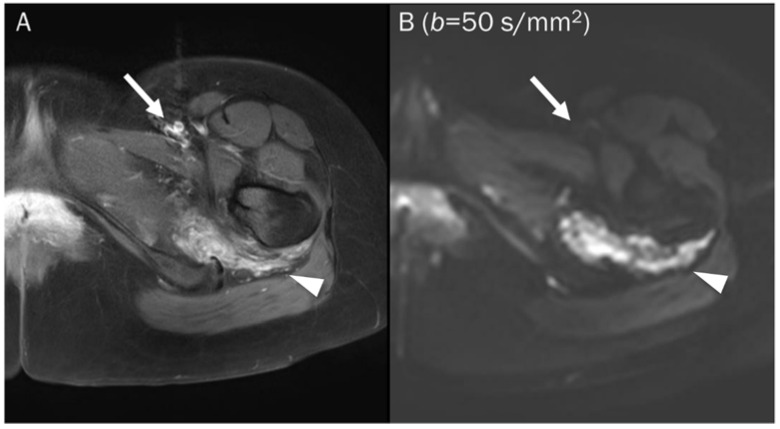
A 25-year-old female with a soft tissue hemangioma in the proximal thigh. (**A**) Axial T1-weighted enhanced image shows hemangioma (arrowhead) at the quadratus femoris muscle and femoral artery and vein (arrow) at the inguinal area. (**B**) DWI with a *b*-value of 50 s/mm^2^ image still exhibits a signal of the hemangioma (arrowhead) due to slow blood flow within the vessels, while the femoral artery and vein (arrow) are invisible due to fast-flowing blood within the vessels, creating a “black blood” effect.

Lower *b*-values tend to have a higher perfusion-related contribution, causing free water to appear brighter due to intrinsic T2-weighting, which can affect the ADC value. Therefore, many protocols exclude the low *b*-value of 0 and choose a *b*-value of 50 s/mm^2^ instead [78]. In low *b*-value (50–100 s/mm^2^) images, free water, such as fast-flowing blood within vessels and cerebrospinal fluid, exhibit significant signal attenuation, often referred to as “black blood” images (Figure 4). This is because water molecules move a relatively large distance during the application of the diffusion-sensitizing gradients [2]. The main contributing factor changes from perfusion to diffusion with *b*-values in the range of 100–300 s/mm^2^ [79]. A *b*-value of at least 500–800 s/mm^2^ is required to separate diffusion from perfusion-related contributions [77]. Although there is no consensus on how many *b*-values should be used when imaging the musculoskeletal system, many recent protocols in the literature suggest three *b*-values (e.g., 50, 400, 800 s/mm^2^) [78,80].

Point: Lower *b*-values have a higher perfusion-related contribution, which can affect the ADC value, so many protocols exclude the low *b*-value of 0 and choose a *b*-value of 50 s/mm^2^ instead, with a *b*-value of at least 500–800 s/mm^2^ required to separate diffusion from perfusion-related contributions.

### 3.2. ADC Value Generation

The ADC value (usually represented by μm^2^/s or × 10^−3^ mm^2^/s) quantifies the gradual loss of signal in a tissue of interest as demonstrated by DWI with increasing diffusion-sensitizing gradient strengths or *b*-values [77]. The ADC value is mathematically defined as the slope of a line that represents a logarithmic decrease in signal intensity between two or more *b*-values, using a monoexponential fit [63]. ADC values are generated on a pixel-by-pixel basis and can measure minimum, maximum, and mean ADC values for interest of region [63]. Although the mean ADC value is commonly used, the minimum ADC value is more correlated with histologic findings [69]. Restricted diffusion leads to an increased signal on DWI with low ADC values, reflecting highly cellular microenvironments with the term “restricted diffusion”, while free diffusion leads to a decreased signal on DWI and increased ADC values, reflecting paucicellular regions with the term “unrestricted or free diffusion” [63]. Therefore, DWI and ADC values provide a quantitative evaluation of cellularity at the molecular level.

Point: ADC values provide quantitative evaluation of cellularity at the molecular level, reflecting restricted diffusion in highly cellular regions and unrestricted or free diffusion in paucicellular regions.

### 3.3. DWI Protocols

DWI is a non-contrast sequence and should be performed prior to the administration of intravenous contrast agents. The single-shot echoplanar spin-echo T2-weighted sequence is dominant in current clinical use of the musculoskeletal system due to its rapid data acquisition, less sensitivity to patient motion, and high signal-to-noise ratio [63]. Unfortunately, this sequence is prone to magnetic susceptibility artifacts, particularly at tissue interfaces such as air, bone, and soft tissue, and to geometric distortions caused by eddy currents in a large field of view [81].

Point: DWI is a non-contrast sequence that should be performed before the administration of intravenous contrast agents, and it is prone to magnetic susceptibility artifacts and geometric distortions.

## 4. DWI Interpretation with ADC Map

Qualitative and quantitative analysis of diffusivity on DWI are both important. In qualitative analysis, it is important to recognize the significance of *b*-values, while in quantitative analysis, one should be aware of the principles of ADC measurement.

### 4.1. Qualitative Analysis

**Figure 5:**
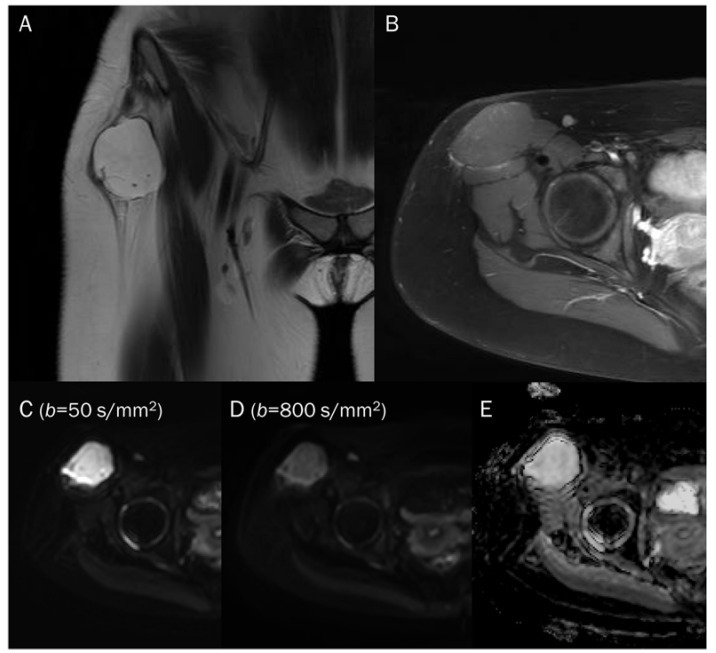
A 15-year-old female with intramuscular myxoma in the sartorius. (**A**) Coronal T2-weighted image shows a lobulated mass with T2 high signal intensity in the sartorius muscle. (**B**) Axial T1-weighted enhanced image shows peripheral and septal enhancement in the mass. (**C**,**D**) DWI with *b*-values of 50 and 800 s/mm^2^ exhibit progressive signal loss of the tumor with (**E**) no reduction in the ADC map, suggesting “free” diffusivity.

**Figure 6:**
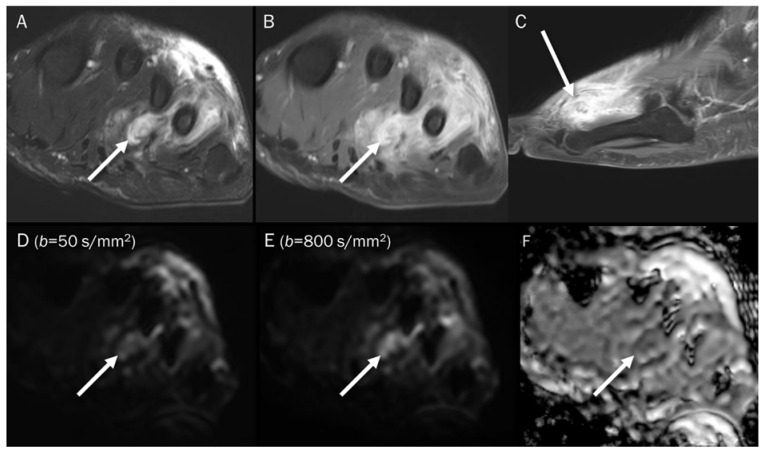
A 39-year-old male with foot dorsum pain. (**A**) Axial T2-weighted fat-suppressed image shows soft tissue swelling at the dorsal surface of the foot and interosseous muscle (arrow). (**B**,**C**) Axial and sagittal T1-weighted enhanced images show heterogeneous enhancement at the affected areas (arrows), suggesting phlegmon or abscess. (**D**,**E**) DWI with *b*-values of 50 and 800 s/mm^2^ exhibit progressive signal increase in the interosseous lesion (arrows) with (**F**) a reduced signal in the ADC map, suggesting “restricted” diffusivity, interpreted as an abscess.

Qualitative analyses rely on the subjective visual interpretation of signal loss as diffusion weighting increases, which can lead to poor reproducibility [82]. Based on a qualitative assessment, diffusivity can be categorized as “free” or “restricted” by visual assessment. Hypocellular tissues exhibit a characteristic of “free” diffusivity (Figure 5), which is indicated by a gradual and exponential decrease in signal intensity as the gradient strengths/*b*-values are progressively increased on DWI [69]. This signal loss is due to the inability of mobile protons to refocus within a hypocellular environment, leading to high measured ADC values. Diffusivity of hypercellular/malignant tissues is “restricted” (Figure 6). This is characterized by a static elevated signal with increasing gradient strengths on DWI and a low measured ADC. [68].

Point: Qualitative analysis of DWI relies on subjective visual interpretation of signal loss, with “free” diffusivity indicating hypocellular tissues and “restricted” diffusivity indicating hypercellular/malignant tissues.

### 4.2. Quantitative Analysis

**Figure 7:**
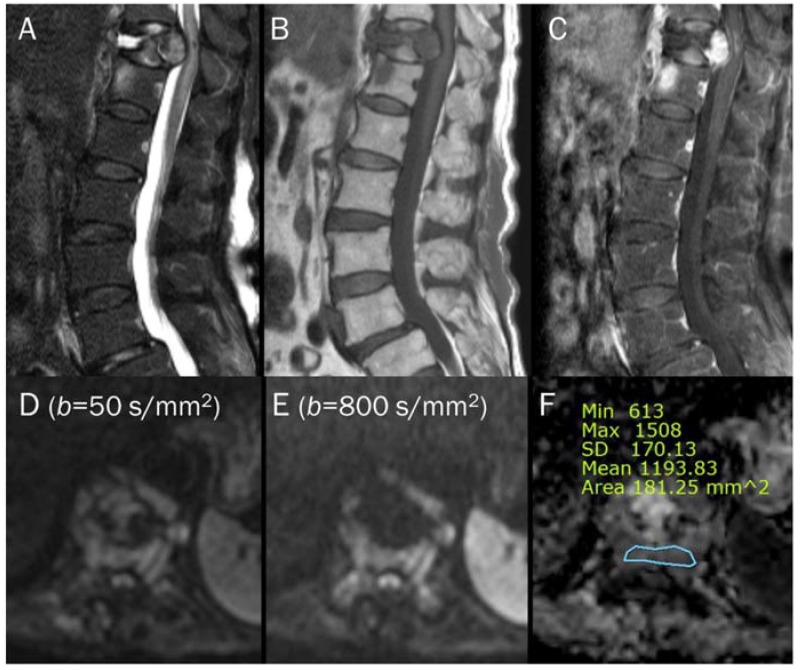
An 87-year-old female identified as pathologic compression fracture with renal cell carcinoma at 11th thoracic vertebra. Conventional sagittal MRIs (**A**: T2-weighted fat-suppressed, **B**: T1-weighted, **C**: T1-weighted enhanced) show severe vertebral collapse with posterior bulging contour, as well as frame-like enhancement around the fluid cleft. (**D**,**E**) DWI with *b*-values of 50 and 800 s/mm^2^ exhibit progressive signal increase in the enhancing cellular portion with (**F**) a reduced signal in the ADC map with mean ADC value of 1193.83 μm^2^/s, suggesting “restricted” diffusivity and interpreting it as bone metastasis. Note that the largest ROI is selected to encompass most of the lowest signal intensity on the ADC map.

In quantitative analyses, the lesion’s minimum, maximum, and mean ADC values are typically measured within a defined region of interest (ROI). Although the minimum ADC values have been found to demonstrate the strongest correlation with histology, the mean ADC is conceptually straightforward and more widely used [2,63]. It is noteworthy that the variability in constructing an ROI on the ADC map due to the absence of standardized methodologies can result in inconsistencies in image analysis and interpretation among different readers [63]. There are varying methodologies regarding whether the ROI should encompass the entire tumor or solely the regions exhibiting the lowest values [63]. Subhawong et al. [63] suggested using circular or elliptic ROIs to cover the largest possible area of the tumor while excluding adjacent bone or soft tissues. This is typically done on images where the tumor exhibits the lowest ADC, indicating the presence of more cellular tissue. The minimum and mean ADC values are then measured [63]. Although there is currently no agreement on the optimal size of the ROI for ADC measurement, it may be most practical and reliable to select ROIs covering both the areas with the lowest signal intensity by qualitative assessment of the ADC map and the largest spatial extent of the lesion to establish a consistent and easily implementable protocol for clinical use (Figure 7) [77]. To avoid obtaining inaccurate ADC values, it is necessary to be cautious and avoid the foci of the mineral, hemorrhage, cortex, and macroscopic fat [77].

Point: Quantitative analyses of ADC values typically measure the lesion’s minimum, maximum, and mean ADC values within a defined region of interest.

## 5. Pitfalls of DWI and ADC Map

The specificity of the ADC value is reduced when the T2 signal intensity of the lesion is markedly hypointense or hyperintense [73]. It is essential to compare DWI and ADC in conjugation with conventional MRI.

### 5.1. T2 Shine-through Effect

**Figure 8:**
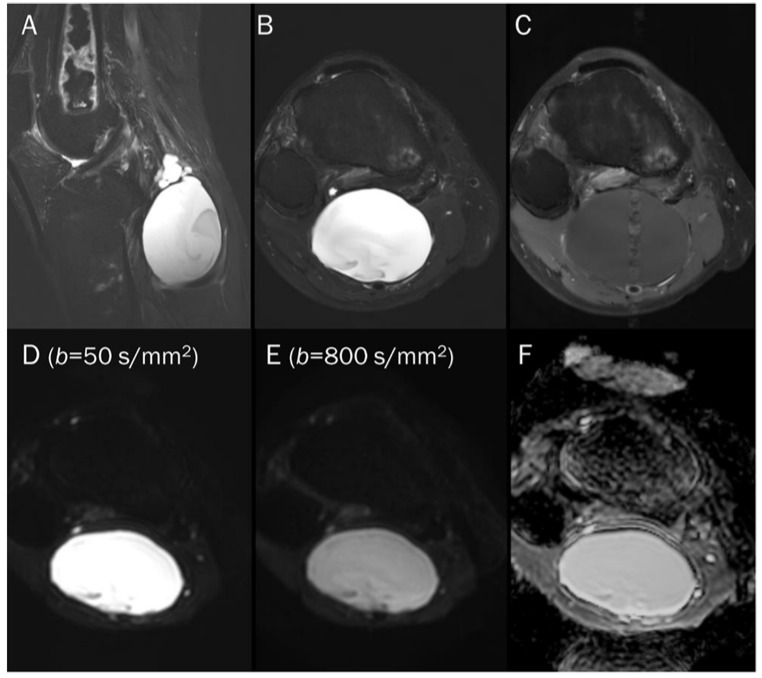
A 55-year-old male with a ganglion cyst in the popliteal fossa. (**A**) Sagittal and (**B**) axial T2-weighted fat-suppressed images show a mass with a bright signal intensity mass in the popliteal fossa. (**C**) Axial T1-weighted enhanced image shows peripheral thin wall enhancement. (**D**,**E**) DWI with *b*-values of 50 and 800 s/mm^2^ exhibit persistent hyperintensity with (**F**) no reduction in the ADC map, suggesting a T2 “shine-through” effect, possibly due to a high T2 relaxation time.

It is important to understand the concept of the T2 shine-through effect. When a lesion contains a significant amount of fluid, there is no restriction of diffusion, and thus no reduction in ADC values, despite the high intensity of DWI due to the long T2 relaxation time [69]. As a result, such tissues appear bright on both DWI and ADC maps (Figure 8). If DWIs are obtained using a single *b*-value, it may not be possible to generate ADC maps, which can lead to potential pitfalls during interpretation. There is a risk of misinterpreting fluid-containing lesions in areas with restricted diffusion by qualitative evaluation only (pseudo-high on DWI with high *b*-value) [68,83]. To avoid such pitfalls, it is recommended to acquire DWI with a minimum of two diffusion weightings.

Point: T2 shine-through effect cause false positive findings due to the long T2 relaxation time of fluid, leading to potential misinterpretation of fluid-containing lesions as areas with restricted diffusion, and acquiring DWI with a minimum of two diffusion weightings is recommended to avoid this pitfall.

### 5.2. T2 Black-out Effect

**Figure 9:**
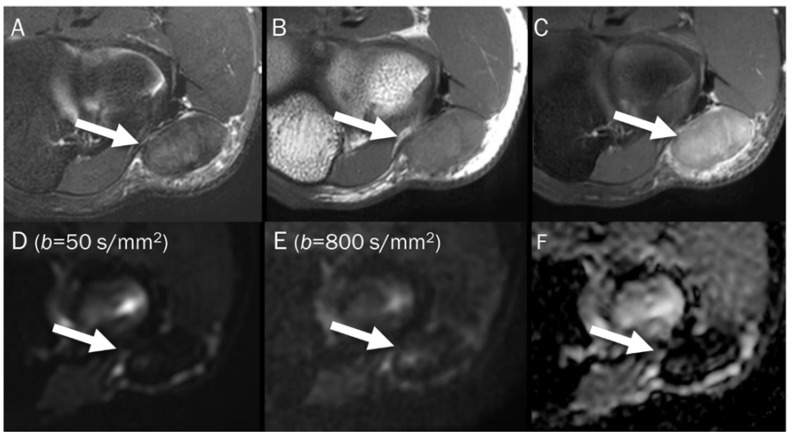
A 46-year-old male with a hematoma on his elbow caused by being struck with a baseball three months ago. Conventional axial MRIs (**A**: T2-weighted, **B**: T1-weighted, **C**: T1-weighted enhanced) show a well-circumscribed mass (arrow) with T2 high and T1 intermediate signal intensity, and heterogeneous enhancement in the elbow. (**D**,**E**) DWI with *b*-values of 50 and 800 s/mm^2^ exhibit persistent low signal (arrow) with different *b*-values, while (**F**) the ADC map shows a signal drop (arrow), indicating T2 “black-out” effect, possibly due to susceptibility artifacts.

The T2 black-out effect, which is the opposite of the T2 shine-through effect, can potentially cause another problem [68]. It is observed when analyzing structures that have a very short intrinsic T2 signal, such as those containing iron or calcium. The low intrinsic T2 signal results in low signal intensity with different diffusion weightings, which can lead to limitations in the accuracy of ADC maps. Lesions that have a lower water content such as those containing fat, or susceptibility artifacts such as hemorrhages containing deoxyhemoglobin or hemosiderin, are likely to exhibit lower signal intensity on DWI with high *b*-values and lower ADC values (pseudo-low ADC value, Figure 9), misinterpreted as highly cellular tumors [2,68]. Assessing osteosarcoma or osteoblastic bone metastases can be challenging due to the presence of this phenomenon, which can decrease the sensitivity of DWI [68]. To prevent misinterpretation, it is important to thoroughly review and correlate T2-weighted images, DWIs obtained with multiple *b*-values, and the corresponding ADC map.

Point: The T2 black-out effect can lead to potential misinterpretation of lesions with a low intrinsic T2 signal or susceptibility artifacts as highly cellular tumors on DWI with lower ADC values.

## 6. Image Interpretation Guidelines for DWI with ADC Map

**Figure 10:**
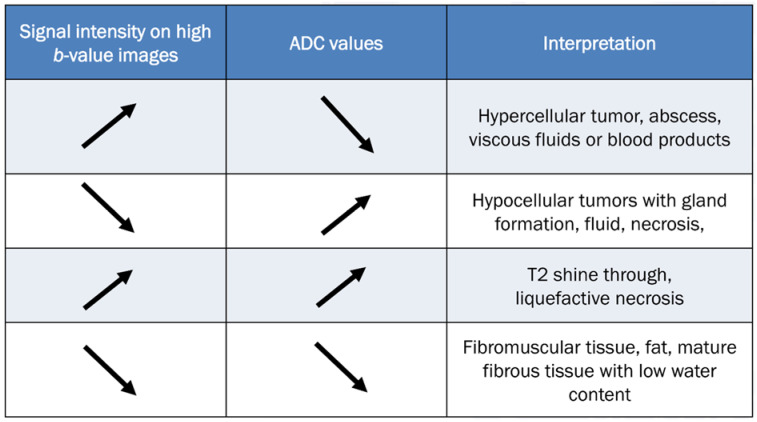
Guidelines for interpreting DWI [2,69]. The table provides interpretation based on the increase in *b*-values and the corresponding ADC map. The direction of the arrow indicates increase and decrease of signal intensity.

To accurately evaluate DWI, it is necessary to visually compare the images acquired using a low *b*-value (T2-weighting), with those obtained using a high *b*-value. Figure 10 provides a comprehensive overview of all possible interpretations of DWI with the ADC map. The top two rows of Figure 10 refer to interpretation related to cellularity, while the bottom two rows refer to potential pitfalls.

In tumorous conditions, it is widely accepted that DWI typically shows low ADC values in malignant aggressive tumors, while benign tumors usually exhibit high ADC values [69]. When interpreting DWI findings, it is important to consider the matrix and appearance of any musculoskeletal tumor on conventional MRI [69]. DWI is valuable for evaluating disease status after treatment, especially in cases such as osteosarcoma and Ewing’s sarcoma, where increasing ADC values from the baseline indicates a good response to chemotherapy [24,84,85].

In non-tumorous conditions, DWI can be diagnostically helpful to differentiate between degenerative and inflammatory diseases, as well as to identify infected areas. Inflammatory conditions such as ankylosing spondylitis presenting sacroiliac and spinal diseases typically exhibit higher ADC values compared to degenerative conditions [69]. Several studies have demonstrated the usefulness of DWI as a technique for monitoring the progress of rheumatic disease after treatment, as patients who respond well to treatment typically show a decrease in ADC values [86]. DWI has also been shown to be effective in detecting infected areas, as the restricted diffusivity observed in infections or abscesses containing protein-rich, viscous liquid can be identified through DWI as restricted diffusion [69].

The clinical applications related to the pitfalls of DWI will be discussed in detail in Section 7.

## 7. Clinical Applications of DWI Pitfalls

If T2 shine-through and T2 black-out effects are interpreted without reference to DWI, there is a risk of misdiagnosing certain musculoskeletal lesions. Lesions that demonstrate the T2 black-out effect (pseudo-low ADC value) may be incorrectly diagnosed as highly cellular tumors when analyzed solely based on the ADC map. Conversely, lesions that demonstrate T2 shine-through (pseudo-high on DWI with high *b*-value) may be misinterpreted as having restricted diffusion when analyzed solely based on the DWI. Therefore, it is important to use a combination of DWI (both low and high *b*-values) and ADC maps for accurate lesion characterization and diagnosis.

### 7.1. Cyst

**Figure 11:**
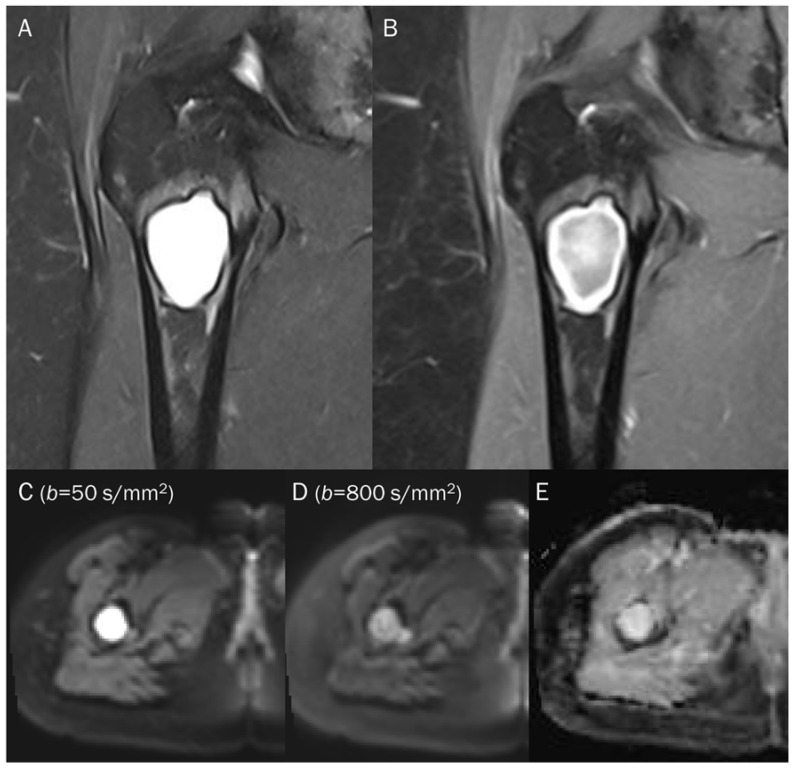
A 38-year-old female with cystic degeneration of fibrous dysplasia in the proximal femur. (**A**) Coronal T2-weighted fat-suppressed and (**B**) T1-weighted enhanced images show an intramedullary, lobulated bony mass with a bright signal intensity mass and heterogeneous enhancement in the proximal femur. (**C**,**D**) DWI with *b*-values of 50 and 800 s/mm^2^ exhibit slight signal loss of the tumor as the *b*-value increases. However, hyperintensity remains in high *b*-values, raising the possibility of “restricted” diffusivity. (**E**) ADC map shows little signal reduction in the ADC map, indicating a T2 “shine-through” effect, possibly due to the high T2 relaxation time of the cystic change of the tumor.

Juxtaarticular ganglia or cysts can typically be diagnosed using only conventional MRI sequences, as these structures do not exhibit internal contrast enhancement [87]. The high ADC values observed in them, attributed to their free water content, have been found to be diagnostically useful. In musculoskeletal tumors, many tumors may appear with cystic change, and their ADC values may be somewhat high. Knowing the presence of the T2 shine-through effect can help distinguish whether the cystic appearance is truly fluid (Figure 8) or a cystic neoplasm (Figure 5), such as myxomas that have homogeneous T2 hyperintensity and may be mistaken for cysts during qualitative analysis [87]. In the case of cystic neoplasms or tumor with cystic changes, even if the ADC is high, some signal loss will appear in the DWI with higher *b*-value images. Additionally, some bone tumors may be overlooked as a hypercellular tumor on DWI alone, but when combined with ADC, it can help predict a cystic change of the underlying bone tumor (Figure 11). When conducting quantitative analysis using ADC measurement, a mean ADC value of greater than 2.5 × 10^−3^ mm^2^/s can achieve a sensitivity of 80% and a specificity of 100% can be achieved in diagnosing cysts and benign cystic lesions [88].

### 7.2. Hematoma

The DWI signal intensity of a hematoma is dependent on its stage and is correlated with the relative amounts of various types of hemorrhagic materials present [89,90]. The changes in DWI signal during the evolution of intracerebral hematomas are well defined (Table 1) [91]. In the hyperacute stage, DWI reveals a hyperintense signal of oxyhemoglobin, and the ADC value is decreased, indicating “restricted” diffusion of water molecules within red blood cells [89,90]. During the late subacute stage, the increased mobility of water in extracellular methemoglobin results in a prolonged hyperintense signal on DWI due to the T2 component of the fluid that contains extracellular methemoglobin. This effect is not affected by uneven susceptibility, and there is a slight increase in ADC value compared to the other stages, indicating ‘slightly restricted’ water diffusion [89,90]. Magnetic susceptibility effects cause other blood clots, such as deoxyhemoglobin (in the acute stage), intracellular methemoglobin (in the early subacute stage), and hemosiderin (in the chronic stage, Figure 9), to display low signal intensity on both T2-weighted images and DWI. The low signal intensity of these hemorrhagic products on T2-weighted images results in unreliable ADC value calculations due to the T2 black-out effect, which creates a pseudo-low ADC value [92].

**Figure 12:**
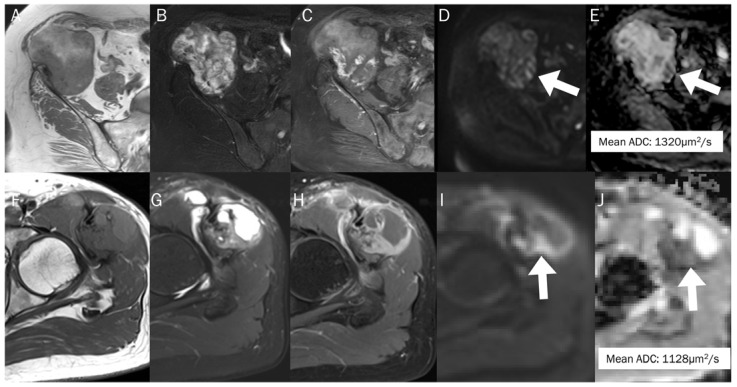
The differentiation between chronic expanding hematoma (**A**–**E**) and hemorrhagic malignant soft tissue tumor (**F**–**J**). Conventional axial MRIs of chronic expanding hematoma (**A**: T1-weighted, **B**: T2-weighted fat-suppressed, **C**: T1-weighted enhanced) show a lobulated mass with heterogeneously high T2 signal intensity and iso-to-high T1 signal intensity, located in the pelvic cavity, containing nodular enhancement. (**D**,**E**) DWI with *b*-value of 800 s/mm^2^ and ADC map exhibit an area of restricted diffusivity (arrow), and the mean ADC is 1320 μm^2^/s. Conventional axial MRIs of hemorrhagic high-grade sarcoma (**F**: T1-weighted, **G**: T2-weighted fat-suppressed, **H**: T1-weighted enhanced) show a lobulated mass with heterogeneously high T2 signal intensity and iso-to-high T1 signal intensity, located in the proximal thigh, containing nodular enhancement. (**I**,**J**) DWI with *b*-value of 800 s/mm^2^ and ADC map exhibit an area of restricted diffusivity (arrow) and the mean ADC is 1128 μm^2^/s.

Many musculoskeletal soft tissue tumors show hemorrhagic changes, which can be difficult to differentiate from hematoma on conventional MRI due to their similar appearances. In such cases, the quantitative use of ADC measurement can aid in differentiation (Figure 12); the mean ADC value of a hematoma is significantly higher than that of hemorrhagic malignant soft tissue tumors (1.55 ± 0.121 × 10^−3^ mm^2^/s vs. 0.92 ± 0.139 × 10^−3^ mm^2^/s) [93].

### 7.3. Benign Bone and Soft Tissue Tumors

**Figure 13:**
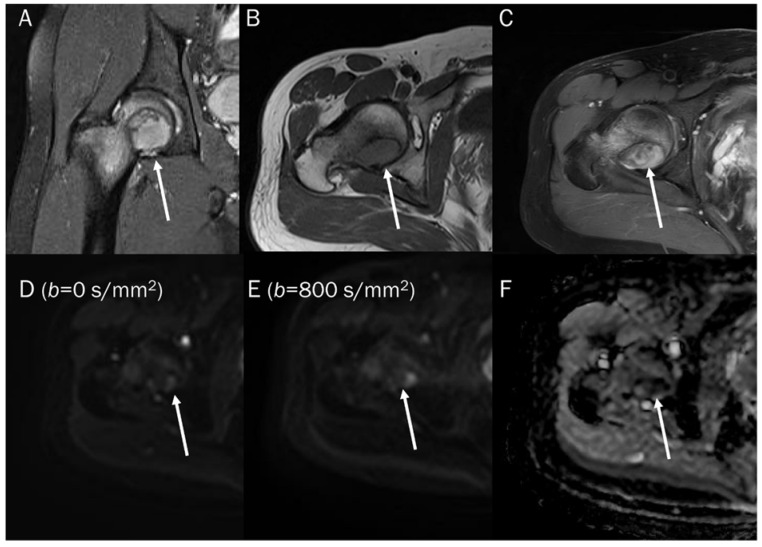
A 30-year-old male with a GCT of bone in the femoral head. Conventional MRIs (**A**: coronal proton-density, **B**: axial T1-weighted, **C**: axial T1-weighted enhanced) show a well-circumscribed mass (arrow) with proton-density high and T1 intermediate signal intensity, as well as heterogeneous enhancement in the femoral head, accompanied by extensive peritumoral edema, raising the possibility of a malignant bone tumor. (**D**,**E**) DWI with *b*-values of 50 and 800 s/mm^2^ exhibit persistent low signal (arrow) with different *b*-values, while (**F**) the ADC map shows a signal drop (arrow), indicating a T2 “black-out” effect, which could be attributed to susceptibility artifacts of hemosiderin.

**Figure 14:**
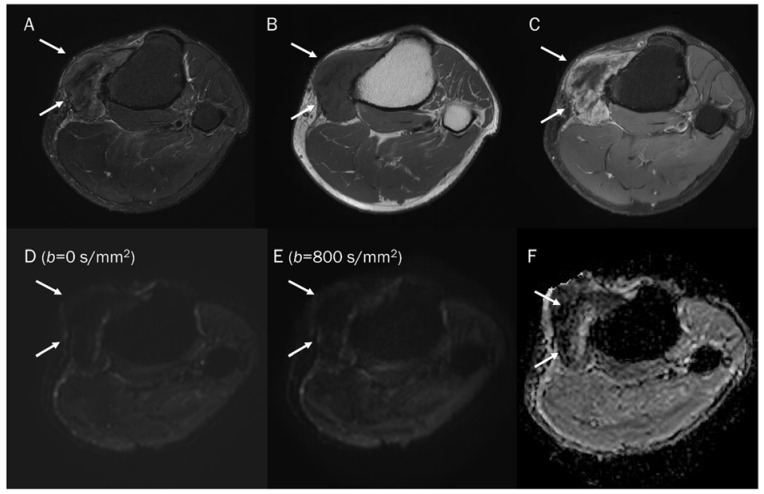
A 47-year-old male with gouty tophi around the knee. Conventional axial MRIs (**A**: T2-weighted fat-suppressed, **B**: T1-weighted, **C**: T1-weighted enhanced) show an infiltrative soft tissue mass (arrow) with heterogeneously high T2 signal intensity and intermediate T1 signal intensity, as well as heterogeneous enhancement at the medial side of the tibia, accompanied by extrinsic erosion of the tibia, raising the possibility of a malignant soft tissue tumor. (**D**,**E**) DWI with *b*-values of 50 and 800 s/mm^2^ exhibit persistent low signal (arrow) with different *b*-values, while (**F**) the ADC map shows a signal drop (arrow), indicating a T2 “black-out” effect, which could be attributed to susceptibility artifacts caused by the mineralization of monosodium urate crystals.

In general, it is commonly believed that malignant bone tumors such as sarcomas and lymphomas tend to have lower ADC values, while benign bone tumors usually have higher ADC values [77,94]. Nevertheless, certain benign bone tumors, such as a non-ossifying fibroma or giant-cell tumor (GCT), may demonstrate the T2 black-out effect, which creates a pseudo-low ADC value that is less than 1.0 × 10^−3^ mm^2^/s [67]. This effect can be ascribed to the dense collage fibers in non-ossifying fibroma and the presence of hemosiderin deposits and other blood products in GCT, which can influence the ADC measurement [65]. Choi et al. [4] reported that certain benign soft tissue tumors, such as those associated with bleeding or schwannoma with secondary degeneration, tenosynovial GCT, and fibroma, can exhibit visual diffusion restriction (the presence of signal drop). In particular, GCT (Figure 13) can sometimes present with peritumoral edema (GCT of bone) or an ill-defined margin (diffuse-type tenosynovial GCT), which can lead to misinterpretation of a malignant tumor when relying solely on conventional MRI [95,96]. Gout with tophi (Figure 14) that contain foci of mineralization can also be erroneously interpreted as a malignant soft tissue lesion [77]. Even with the addition of DWI, if the reader were to only see the ADC map, restricted diffusion could be mistakenly assumed due to the T2 black-out effect. Therefore, it is crucial to examine both the DWI and ADC map to determine if the mass is a certain benign tumor with the T2 black-out effect.

### 7.4. Vertebral Endplate Changes

**Figure 15:**
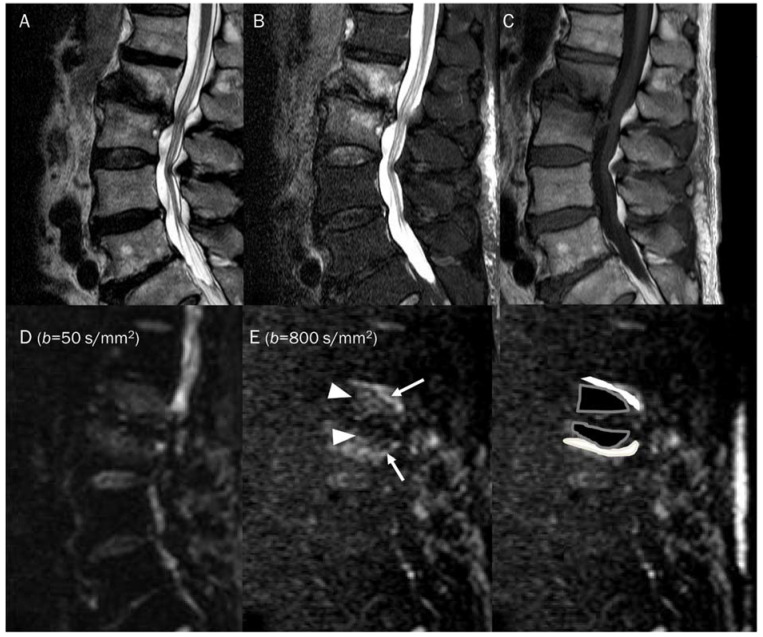
A 81-year-old male with type 1 Modic change in the endplates of L2-3. Conventional sagittal MRIs (**A**: T2-weighted, **B**: T2-weighted fat-suppressed, **C**: T1-weighted) show an indistinct bone marrow edema in the adjoining endplates of L2-3. (**D**,**E**) DWI with *b*-values of 50 and 800 s/mm^2^ exhibit persistent outer high signal bands (arrows) with inner low signal areas (arrowheads), displaying a ‘claw sign’, indicative of type 1 Modic change. The inner low signal areas may be due to the T2 black-out effect caused by fatty marrow.

Degenerative vertebral endplate changes are classified into three groups by Modic. In type 1 Modic change, the presence of vascular granulation tissue is noted, which appears as low signal intensity on T1-weighted images and high signal intensity on T2-weighted images [97]. Histological examination has revealed that these changes are linked to the fissuring of the cartilaginous endplate and increased vascularity within the subchondral bone marrow [98]. Diagnosing type 1 Modic changes in patients with low back pain can be challenging because their MRI features resemble those of spondylodiscitis. Typically, spondylodiscitis causes vertebral marrow edema, which appears as areas of low signal intensity on T1-weighted images and high signal intensity on T2-weighted images [99]. Distinguishing between two conditions, type 1 Modic change and spondylodiscitis, can be relatively easy using DWI. The presence of the ‘claw sign’ on DWI is highly indicative of degenerative change, while its absence is a strong indicator of infectious spondylodiscitis (Figure 15) [100]. The claw sign observed on DWI manifests as paired, well-defined linear areas of high signal intensity. These areas are situated at the interface between normal bone marrow and vascularized bone marrow, which is in close proximity to the affected disc [100]. The crucial point is that the inner portion of the high signal intensities appears lower in signal intensity than the outer part on DWI. This is believed to be caused by a combination of affected fatty bone marrow and granulation tissue. The presence of the fat marrow component results in a low signal intensity within the affected area. Larger lipid-laden cells in fatty marrow, along with bone trabeculae, can impede the movement of extracellular water, leading to a T2 black-out effect [101].

Table 2 summarized the clinical usefulness of DWI pitfalls in musculoskeletal diseases.

## 8. Conclusions

DWI is a functional imaging technique and is becoming increasingly popular as part of multiparametric MRI for evaluating musculoskeletal diseases. DWI provides both qualitative and quantitative information for characterizing not only tumorous diseases but also non-tumorous diseases in musculoskeletal radiology. However, the interpretation of the DWI and the ADC map should take into account the potential pitfalls. For instance, there is a risk of misinterpreting a lesion with a T2 black-out effect as a highly cellular tumor with pseudo-low ADC values. Additionally, there is a risk of misinterpreting fluid-containing lesion as areas with restricted diffusion due to T2 shine-through effect, resulting in pseudo-high signals on DWI with a high *b*-value. Therefore, when assessing ADC values, conventional MRI and DWI must be considered. Careful interpretation of DWI and ADC map, considering the pitfalls, combined with conventional MRI and clinical findings, can enhance diagnostic accuracy, and facilitate the selection of the most suitable management for certain musculoskeletal diseases.

## Figures and Tables

**Table 1:** Signal characteristics of the intracerebral hematoma on DWI and ADC maps.

Stage	Component	Age	T1WI	T2WI	DWI	ADC Map
Hyperacute	Intracellular oxyhemoglobin	<6 h	Iso	Hyper	Hyper	Hypo
Acute	Intracellular deoxyhemoglobin	6–72 h	Iso	Hypo	Hypo	Hypo
Early subacute	Intracellular methemoglobin	3–7 d	Hyper	Hypo	Hypo	Hypo
Late subacute	Extracellular methemoglobin	1–4 w	Hyper	Hyper	Hyper	Hypo-to-iso
Chronic	Hemosiderin	>1 m	Hypo	Hypo	Hypo	Hypo

**Table 2:** Summary of DWI pitfalls in musculoskeletal diseases.

Lesions	Pitfalls	Content
Benign cyst	T2 shine-through	Free water
Hematoma		
Acute stage	T2 black-out	Deoxyhemoglobin
Early subacute stage	T2 black-out	Intracellular methemoglobin
Chronic stage	T2 black-out	Hemosiderin
Benign tumors		
Non-ossifying fibroma	T2 black-out	Collagen fibers
Giant-cell tumor	T2 black-out	Hemosiderin
Gouty tophi	T2 black-out	Monosodium urate crystal
Modic type 1 vertebral endplate change	T2 black-out	Lipid laden cells

## Data Availability

Not applicable.
